# Outcomes of Six-Dose High-Dose Cytarabine as a Salvage Regimen for Patients with Relapsed/Refractory Acute Myeloid Leukemia 

**DOI:** 10.1155/2017/6464972

**Published:** 2017-05-14

**Authors:** Brandi Anders, Lauren Veltri, Abraham S. Kanate, Alexandra Shillingburg, Nilay Shah, Michael Craig, Aaron Cumpston

**Affiliations:** ^1^Department of Pharmacy, West Virginia University Medicine, Morgantown, WV, USA; ^2^Section of Hematology/Oncology, Department of Internal Medicine, West Virginia University, Morgantown, WV, USA; ^3^Osborn Hematopoietic Malignancy and Transplantation Program, MBRCC, West Virginia University, Morgantown, WV, USA

## Abstract

Relapsed/refractory acute myeloid leukemia (RR-AML) is associated with poor prognosis and long-term disease-free survival requires allogeneic hematopoietic cell transplantation (allo-HCT). Limited data exists, regarding the optimal regimen to obtain remission prior to allo-HCT. Single agent high-dose cytarabine (10–12 doses administered every 12 hours) has been previously used as induction therapy. Six-dose high-dose cytarabine (HiDAC-6), commonly used as a consolidation regimen, has never been evaluated as induction therapy. We present a retrospective review of 26 consecutive patients with RR-AML receiving single agent cytarabine 3 g/m^2^ intravenously every 12 hours on days 1, 3, and 5 for a total of six doses (HiDAC-6). Median follow-up for surviving patients was 10.4 months (range 1.6–112.2 months). Complete remission was obtained in 62% (54% CR and 8% CRi) of the patients. The median relapse-free survival (RFS) was 22.3 months (range 0.7–112 months), event-free survival (EFS) was 4.7 months (range 0.5–112 months), and the overall survival (OS) was 9.6 months (range 1–112 months). Thirty-five percent of patients were able to subsequently proceed to allo-HCT. Treatment-related toxicities included neutropenic fever (38%), infection (35%), neurotoxicity (8%), and skin toxicity (8%). This is the first study to demonstrate HiDAC-6 as an active treatment option for younger patients with RR-AML which can effectively serve as a bridge to allo-HCT without significant toxicity.

## 1. Introduction

Acute myeloid leukemia (AML) remains the most common form of acute leukemia among adults and is responsible for the largest number of deaths from leukemia in the United States [1]. Standard front-line induction therapy achieves complete response (CR) rates of 60–80%; however these remissions are often transient and the majority of patients recur within one to three years after diagnosis [2–4]. Patients who do not enter remission or relapse within 6 months of achieving CR have relatively low response rates to reinduction therapy [5–7]. There are several salvage regimens that can be considered for patients with relapsed/refractory AML (RR-AML), with limited data to guide selection of the optimal regimen. Single agent high-dose cytarabine is established for consolidation of AML [8, 9] and has also been studied in the relapsed/refractory setting either as monotherapy or in combination with another agent, frequently an anthracycline [10, 11]. The maximum tolerated dose was found to be 3 g/m^2^ every 12 hours for six days for a total of 12 doses (HiDAC-12) with toxicities associated with higher dose and extended duration including conjunctivitis and liver, dermatologic, and CNS toxicity [10]. HiDAC-12 demonstrated improved response rates with the higher dose and complete remission rate of 63% in patients with RR-AML [10]. The HiDAC regimen utilized in the consolidation phase of treatment has been reduced to 6 doses (HiDAC-6) to reduce cerebellar toxicities. The current era HiDAC-6 regimen has never been evaluated as a salvage regimen for RR-AML. We conducted a retrospective evaluation of response rates and toxicities of HiDAC-6 at our institution in the RR-AML patient population.

## 2. Methods

This retrospective study was conducted at West Virginia University Hospitals. Patients included were admitted to the inpatient adult hematologic malignancy service between June 2001 and July 2015 for RR-AML. All patients received induction chemotherapy with single agent cytarabine 3 g/m^2^ intravenously (IV) every 12 hours on days 1, 3, and 5 for a total of 6 doses (HiDAC-6). This study was approved by the Protocol Review and Monitoring Committee and the Institutional Review Board.

The primary outcome was complete remission [CR + CR with incomplete remission (CRi)] after reinduction with HiDAC-6. The secondary outcomes included overall survival (OS), event-free survival (EFS), and relapse-free survival (RFS), evaluating CR rates stratified by cytogenetic abnormalities, ability to undergo allogeneic transplant, and regimen-related toxicities. Disease responses (CR, CRi, OS, EFS, and RFS) and genetic risk groups were defined according to recommendations published by Döhner et al. [3]. Toxicity assessments were defined according to common terminology criteria of adverse events (CTCAE) version 4.03 [12]. Platelet and neutrophil recovery were defined as platelet count greater than 100 × 10^9^/L and absolute neutrophil count (ANC) > 1.0 × 10^9^, respectively. All primary and secondary outcomes were reported with the use of descriptive statistics.

### 2.1. Supportive Care

All patients received antibacterial (levofloxacin), antifungal (fluconazole/posaconazole), and antiviral (acyclovir) prophylaxis from the start of chemotherapy until resolution of neutropenia. Other supportive care received by each patient included standard antiemetic prophylaxis with 5HT3 receptor antagonists, dexamethasone eye drops to prevent conjunctivitis, and white blood cell growth factor support. Cerebellar assessments were performed prior to each dose of cytarabine. Routine transfusion support included red blood cell and platelet infusions administered for hemoglobin <8 g/dL and platelet count <10 × 10^9^/L, respectively.

## 3. Results

Baseline characteristics of the patients are presented in Table 1. A total of 26 patients with a median age of 46 (range 20–58) were included in the study. The majority of patients had a diagnosis of de novo AML and had received a median of one (range 1–4) prior therapy. Eighty-eight percent of patients had previously received 7 + 3 (cytarabine + anthracycline) and 31% had prior HiDAC. At the time of treatment with HiDAC-6 reinduction, 69% (*n* = 18) had refractory disease and 31% (*n* = 8) of patients had relapsed from prior treatment. Two patients had undergone prior allogeneic hematopoietic cell transplant (allo-HCT).

Median follow-up for surviving patients was 10.4 months (range 1.6–112.2 months). The complete response rate for the 26 patients included in the study was 62% (*n* = 16) and included CR in 54% (*n* = 14) and CRi in 8% (*n* = 2). The median RFS, EFS, and OS were 22.3 months (range 0.7–112 months), 4.7 months (range 0.5–112 months), and 9.6 months (range 1–112 months), respectively. Stratified according to the genetic risk groups, 73% (*n* = 11) of patients in the favorable/intermediate I risk group achieved CR, whereas 45% (*n* = 5) in the intermediate II/poor risk group achieved CR (Table 2). Thirty-five percent (*n* = 9) of patients were able to subsequently proceed to allo-HCT.

All patients in the study experienced grade 4 thrombocytopenia and neutropenia. Median time to neutrophil and platelet recovery was 23 days (range 15–53 days) and 23 days (range 13–53 days), respectively. The most common nonhematological adverse event associated with HiDAC-6 was neutropenic fever (38%). Nine patients (35%) had confirmed infection, most commonly fungal pneumonia (15%) or bacteremia (15%). Additional nonhematological toxicities and associated frequencies are listed in Table 3.

## 4. Discussion

To the best of our knowledge, this is the only report evaluating HiDAC-6 as a salvage reinduction regimen for RR-AML. With a CR rate of 62%, HiDAC-6 may be considered an effective salvage regimen and demonstrates similar CR rates compared to previously reported data evaluating more intense HiDAC-12 regimen (CR = 63%) and combination chemotherapy with cytarabine (CR = 30–60%) [7, 13–15].

Clinical impact of cytogenetic abnormalities and gene mutations on AML outcomes was not well described at the time of most prior single agent HiDAC salvage regimens. We had cytogenetic data on all patients and molecular mutations on 58% (*n* = 15). Although limited by a small sample size, reasonable CR rates were noted across all risk-categories of AML. However, as expected, better responses were noted in those in “better-risk” groups. While only 40% of those with poor risk cytogenetics achieved CR, 80% of patients in favorable risk group entered CR with HiDAC-6. These response rates are especially noteworthy considering the majority of the patients had refractory disease (69%, *n* = 18), which is uniformly associated with poor outcomes [3, 16]. With HiDAC-6, 67% (*n* = 12) of these patients were able to proceed to allo-HCT. Among those with relapsed AML 31% (*n* = 8), CR was noted in 50% (*n* = 4) and 2 proceeded to allo-HCT. Salvage therapy with HiDAC-6 appears to be a feasible bridge to allo-HCT in patients with RR-AML.

HiDAC-6 was well tolerated, with primary nonhematologic complications being neutropenic fever and fungal pneumonia. Infectious complications are anticipated due to prolonged neutropenia associated with AML induction regimens and prophylactic antimicrobials and supportive care are vital components of therapy. Specifically, the high rate of fungal pneumonia (*n* = 4) highlights the need for mold prophylaxis in these patients. All 4 patients with fungal pneumonia were prior to the availability of posaconazole, which has been shown to reduce fungal infections in this patient population [17]. In the Cancer and Leukemia Group B (CALGB) study evaluating HiDAC-6 consolidation, 71% of patients required hospitalization for neutropenic fever or other complications [8]. The CNS toxicity in the CALGB study was 12% with a higher rate (32%) among those older than 60 years of age [8]. Two patients (8%) in our study developed cerebellar toxicity with prompt resolution of symptoms with discontinuation of therapy.

## 5. Conclusions

Albeit limited by a small sample size and retrospective design, HiDAC-6 may be considered an effective salvage reinduction regimen for younger patients with RR-AML and can be utilized as a bridge to allo-HCT.

## Figures and Tables

**Table 1:** Patient characteristics.

Characteristics	*N* = 26
Median age, years (range)	46 (20–58)
Male gender, *n* (%)	16 (62%)
Median BMI (range)	28.2 (18.4–39.6)
AML diagnosis, *n* (%)	
(i) De novo	23 (88%)
(ii) Treatment-related	3 (12%)
Prior MDS, *n* (%)	3 (12%)
ECOG performance status, median (range)	1 (0–2)
Disease status, *n* (%)	
(i) Refractory	18 (69%)
(ii) Relapsed	8 (31%)
Prior therapies, median (range)	1 (1–4)
Prior treatments	
7 + 3	23 (88%)
HiDAC	8 (31%)
Etoposide/mitoxantrone	2 (8%)
Prior allo-HCT, *n* (%)	2 (8%)
Genetic risk group [3], *n* (%)	
Favorable	5 (19%)
Intermediate I	10 (39%)
Intermediate II	6 (23%)
Poor risk	5 (19%)

BMI: body mass index, AML: acute myeloid leukemia, MDS: myelodysplastic syndromes, ECOG: Eastern Cooperative Oncology Group, 7 + 3: cytarabine + anthracycline chemotherapy, HiDAC: high-dose cytarabine, and HCT: hematopoietic cell transplant.

**Table 2:** Response stratified by genetic risk group.

Risk group	CR + CRi *N* (%)
Favorable (*N* = 5)	4 (80)
Intermediate I (*N* = 10)	7 (70)
Intermediate II (*N* = 6)	3 (50)
Poor (*N* = 5)	2 (40)

CR: complete response; CRi: complete response with incomplete count recovery.

**Table 3:** Nonhematological adverse events.

Toxicity	Number of patients (%)
Liver toxicity (Grade 2)	1 (4)

Mucositis	
(i) Grade 1 (mild)	1 (4)
(ii) Grade ≥ 2 (moderate-severe)	2 (8)

Cerebellar toxicity	2 (8)

Neutropenic fever	10 (38)

Infection	
(i) Fungal pneumonia	4 (15)
(ii) Pneumonia, unknown pathogen	1 (4)
(iii) Bacteremia	4 (15)
(iv) Cellulitis	1 (4)

Skin toxicity	2 (8)

Other toxicities: nausea (1) and pericardial effusion (*n* = 1)	
